# The Preliminary Effects of Capsaicin Supplementation on Cerebrovascular Function and Cognition in Middle-Aged and Older Adults Who Are Overweight or Obese

**DOI:** 10.3390/nu18213613

**Published:** 2026-10-30

**Authors:** Tammy Thornton, Emma Kim, Tahnee Downs, Dean Mills, Edward Bliss

**Affiliations:** 1 School of Health, Psychological and Medical Sciences, University of Southern Queensland, Ipswich, QLD 4305, Australia; tammy.thornton@unisq.edu.au (T.T.); tahnee.downs@unisq.edu.au (T.D.); dean.mills@unisq.edu.au (D.M.); 2 Institute for Health, University of Southern Queensland, Ipswich, QLD 4305, Australia; 3 Rural Clinical School, Medical School, Faculty of Health, Medicine and Behavioural Sciences, The University of Queensland, Toowoomba, QLD 4350, Australia; 4 School of Business, Law, Humanities and Pathways, University of Southern Queensland, Toowoomba, QLD 4350, Australia; 5 First Nations Portfolio, University of Southern Queensland, Toowoomba, QLD 4350, Australia

**Keywords:** capsaicin, cognition, cerebrovascular circulation, cerebrovascular function, neurovascular coupling, obesity, ageing

## Abstract

**Background/Objectives:** Cerebrovascular function and cognition decline with age and may be further exacerbated by obesity. Capsaicin may improve cerebrovascular function and cognition, although evidence in humans is lacking. We investigated the effects of 12 weeks of capsaicin supplementation on cerebrovascular function and cognition in middle-aged and older adults who were overweight or obese. **Methods:** Forty participants were randomly allocated to receive 200 mg/day of Capsifen^®^ or a placebo, with 34 completing the intervention (*n* = 17 in both). Before and after supplementation, transcranial Doppler ultrasonography was used to measure the cerebrovascular responsiveness (CVR) to physiological (hypercapnia, 5% carbon dioxide) and cognitive stimuli, while cognition was assessed using a comprehensive cognitive battery. The trial was prospectively registered (ACTRN12623000556640). **Results:** Total CVR to cognitive stimuli increased following Capsifen^®^ supplementation (18 ± 3% to 21 ± 1%) and decreased in the placebo group (21 ± 4% to 19 ± 3%; *p* = 0.0002). Total cognition increased following Capsifen^®^ supplementation (98 ± 18 to 107 ± 18) compared with the placebo (105 ± 13 to 106 ± 10; *p* = 0.0085). Capsifen^®^ did not alter CVR to hypercapnia (30 ± 18% to 44 ± 27% vs. 39 ± 23% to 32 ± 16%; *p* = 0.5981). Working memory, executive function and exercise performance also improved, while waist circumference and waist-to-hip ratio decreased. **Conclusions:** Twelve weeks of Capsifen^®^ supplementation improved total cognition and CVR to cognitive stimuli but not CVR to hypercapnia. These findings provide preliminary evidence that chronic capsaicin supplementation may improve cognition and neurovascular coupling in humans.

## 1. Introduction

Ageing is the leading unmodifiable risk factor of cognitive decline [1,2,3], while obesity is a modifiable risk factor that can exacerbate age-related cognitive decline [4,5]. Ageing and obesity both impair endothelial function through hormonal changes, chronic low-grade systemic inflammation and increased oxidative stress [6,7]. Oxidative stress and inflammation can uncouple endothelial nitric oxide synthase (eNOS), thus reducing the production and protective functions of nitric oxide (NO) [8,9]. NO contributes to the regulation of vascular tone and vascular remodelling, and reduced NO bioavailability predisposes to endothelial dysfunction and reduced cerebrovascular function [1,2,3]. Therefore, the reductions in cerebrovascular function and cognition observed during normal ageing may be exacerbated by obesity [1,10]. These reductions can predispose individuals to neurodegenerative diseases, such as dementia, which are major causes of functional disability and reduced quality of life [11].

There is no clear evidence that pharmaceutical or cognitive training interventions are effective in slowing or preventing cognitive decline [8,9]. Dietary and lifestyle interventions, including exercise training, can improve cerebrovascular function and cognition [12,13]. However, these strategies can have low compliance, particularly among individuals already experiencing cognitive decline [9,14]. Therefore, it is important to investigate cost-effective, evidence-based preventative strategies that may have greater compliance and improve the underlying risk factors associated with reduced cerebrovascular function and cognition.

Capsaicin is a pungent molecule found in plants belonging to the *Capsicum* genus [15]. In animal models, capsaicin reduces chronic low-grade systemic inflammation and oxidative stress, which may ultimately improve cerebrovascular function and cognition [16,17,18]. Capsaicin has also been shown to reduce adiposity and obesity-related pro-inflammatory cytokines including high-sensitivity C-reactive protein (hs-CRP), interleukin-6, and tumour necrosis factor-α in animal models [19]. Therefore, capsaicin may reduce adiposity and systemic inflammation, thereby improving eNOS function and NO synthesis in animal models [19]. A systematic review of eight animal studies reported improvements in spatial and working memory, learning and emotional behaviours in rodents following acute or chronic capsaicin treatment [20]. To our knowledge, no human trials have investigated the effects of capsaicin supplementation on cerebrovascular function and cognition. This may be due to pungency-related side effects, such as gastrointestinal irritation, which make it difficult to evaluate the effects of capsaicin supplementation in humans [21]. Readily available capsaicin supplements, such as Capsifen^®^, use sustained-release encapsulation to prevent degradation before reaching the small intestine [22]. This provides a safe and tolerable method of administering capsaicin in human trials while reducing the pungency-related side effects commonly associated with oral capsaicin intake.

To address this gap, we investigated the effects of 12 weeks of supplementation with 200 mg of oral Capsifen^®^ on cerebrovascular function and cognition in middle-aged and older adults who were overweight or obese. We hypothesised that, compared with a placebo, Capsifen^®^ would: (1) increase the cerebrovascular responsiveness (CVR) to both physiological and cognitive stimuli, representing an improvement in cerebrovascular function, and (2) improve total cognitive capacity, therefore representing an improvement in total cognition.

## 2. Methods

### 2.1. Participants

The inclusion criteria were adults aged between 50 and 80 years with a body mass index (BMI) ≥ 25 kg/m^2^. Participants were recruited from the Darling Downs, West Moreton, South West and Burnett regions of Queensland, Australia, between August and September 2023. Recruitment was undertaken through an approved media campaign incorporating print media, radio, television and social media. Recruitment across these regions provided opportunities for participation among regional and rural populations, including those from lower socioeconomic areas, who are commonly underrepresented in clinical trials [23]. The exclusion criteria were: age under 50 years or over 80 years; BMI < 25 kg/m^2^; current cigarette smoking; blood pressure ≥ 180/100 mmHg; use of oral anticoagulants; a history of or current cardiovascular disease, including myocardial infarction, heart failure or angina; cerebrovascular disease, including stroke; neurological disease, including diagnosed cognitive impairment or neurodegenerative disease; allergy to any treatment or placebo ingredient; consumption of more than one chilli-containing meal or serving of chilli per day; and daily use of a chilli-containing supplement. Participants who were taking medications, including statins and antihypertensive medications, were eligible provided they had remained on a stable treatment regimen, and the medication did not contraindicate participation in the study. A customised health and wellbeing screening questionnaire was used to determine whether participants met the inclusion and exclusion criteria, including their medical history and usual consumption of chilli-containing foods and supplements. The study procedures were approved by the University of Southern Queensland Human Research Ethics Committee (H22REA177), which operates in accordance with the Declaration of Helsinki. The study was prospectively registered with the Australian New Zealand Clinical Trials Registry (ACTRN12623000556640). All participants provided written informed consent before commencing study procedures.

### 2.2. Experimental Design

The study adopted a multi-site, randomised, double-blind, placebo-controlled design. The intervention lasted 12 weeks. Before and immediately following the intervention, participants attended a laboratory at either the University of Southern Queensland Ipswich or Toowoomba campuses, based on their geographical location. Baseline and post-intervention assessments were conducted at a similar time of day. Participants fasted and abstained from coffee, tea and other stimulants for 12 h before each visit. They were also asked to refrain from moderate- to vigorous-intensity exercise for 24 h before each visit and to take their daily supplements and medications after completion of the study assessments. During each visit, participants underwent anthropometric, cardiovascular, respiratory, exercise performance, handgrip strength, cerebrovascular and cognitive assessments and completed the Profile of Mood States questionnaire (POMS). POMS scores were calculated as previously described [24]. Lower scores for the negative mood states and total mood disturbance indicated better mood, while higher scores for the positive mood state indicated better mood. Participants were also provided with a pathology request form and attended a quality-accredited commercial pathology laboratory (QML Pathology, Brisbane QLD, Australia) for independent blood collection and biomarker testing on the day following each baseline and post-intervention laboratory visit. Biomarker analysis included a general chemistry profile, hs-CRP and a complete lipid profile, as previously described [13,25].

### 2.3. Intervention

The two arms of the study included a treatment (Capsifen^®^) and a placebo group. Participants were randomly allocated to one of the two groups using Altman’s minimisation method, balancing the groups for sex, age and BMI [26]. The baseline characteristics of the two groups are presented in Table 1. Participants in both groups consumed two standardised size 1 vegetable capsules daily (The Capsule Guy, Adelaide, Australia). Each Capsifen^®^ capsule contained 100 mg of Capsifen^®^ (Akay Natural Ingredients Private Limited, Kerala, India; lot number L02511A), providing at least 2.8% capsaicinoids, comprising 1.8% (*w*/*w*) capsaicin, 0.84% (*w*/*w*) dihydrocapsaicin and 0.16% (*w*/*w*) nordihydrocapsaicin within a patented beadlet formulation [22]. The 200 mg/day dose was selected based on previous human research demonstrating the safety and tolerability of this dose of the sustained-intestinal Capsifen^®^ formulation in adults who were overweight [27,28]. The daily dose provided at least 5.6 mg of total capsaicinoids, comprising 3.6 mg of capsaicin, 1.68 mg of dihydrocapsaicin and 0.32 mg of nordihydrocapsaicin. The formulation was designed to mask pungency and reduce the gastrointestinal discomfort associated with conventional oral capsaicin intake [27,28]. Each placebo capsule contained 100 mg of maltodextrin and was colour-matched to the Capsifen^®^ capsules. Therefore, participants consumed a total daily dose of 200 mg of Capsifen^®^ or 200 mg of maltodextrin for 12 weeks. Compliance with the intervention was supported through monthly telephone calls, emails and/or text messages. Compliance was assessed by counting the capsules returned at the completion of the intervention and calculating the proportion of capsules consumed relative to the number prescribed. Capsifen^®^ was purchased independently by the research team, and the manufacturer had no involvement in the study design, conduct, data collection, analysis, interpretation or preparation of the manuscript.

### 2.4. Cerebrovascular Haemodynamics

Cerebrovascular haemodynamics were measured non-invasively using transcranial Doppler ultrasonography (TCD; DopplerBox X; Compumedics DWL, Singen, Germany). Measurements included minimum, maximum and mean middle cerebral artery velocity (MCAv) and CVR to hypercapnia and cognitive stimuli [29]. Participants were fitted with a headpiece housing two 2 MHz TCD ultrasound probes, which were positioned over the left and right transtemporal windows. The middle cerebral arteries were insonated bilaterally at a depth of approximately 40–65 mm using standardised techniques, as previously described [29,30]. Once suitable blood velocity signals were obtained, participants rested quietly in a seated position for 10 min and were asked to refrain from excessive movement. Baseline measurements were subsequently recorded for 30 s before the hypercapnic challenge and each cognitive task.

### 2.5. Cerebrovascular Responsiveness to Hypercapnia

Following baseline recordings, participants underwent a 3 min hypercapnic challenge and were monitored for a further 1 min following removal of the stimulus. The challenge was performed twice, with a 5 min rest period between trials during which participants breathed room air ad libitum. This allowed mean MCAv to return to baseline before repetition of the challenge [29]. During each hypercapnic challenge, participants wore a nose clip and breathed through a two-way non-rebreathing valve (model 2730; Hans Rudolph, Kansas City, MO, USA). The inspiratory port of the valve was connected by 1 m of wide-bore tubing to a 100 L Douglas bag containing carbogen gas comprising 5% carbon dioxide and 95% oxygen (Carbogen 5; BOC, Toowoomba, Australia).

Expiratory flow was measured using a pneumotachograph connected to the expiratory port of the valve (MLT1000L; ADInstruments, Bella Vista, Australia). The pneumotachograph was calibrated using a 3 L syringe before each test, and volume was calculated by numerical integration of the flow signal. End-tidal partial pressure of carbon dioxide (P_ET_CO_2_) was sampled from the expiratory port using a gas analyser (ML206; ADInstruments, Bella Vista, Australia), which was calibrated across the physiological range using gases of known concentration (BOC, Toowoomba, Australia). Flow and P_ET_CO_2_ signals were sampled at 200 Hz using a PowerLab C analogue-to-digital converter (ADInstruments, Bella Vista, Australia), interfaced with a computer and displayed in real time during testing. Data were stored for subsequent offline processing and analysis using LabChart software, version 8 (ADInstruments, Bella Vista, Australia).

### 2.6. Cognition and Cerebrovascular Responsiveness to Cognitive Stimuli

Cognition was assessed using the Trail Making Task Parts A and B and the National Institutes of Health (NIH) Toolbox Cognition Battery, which comprises a battery of validated cognitive assessments [31,32]. An age-adjusted total composite cognitive function score was derived from the NIH Toolbox Cognition Battery [33]. NIH Toolbox scores were automatically computed within the program, thereby minimising potential examiner and scoring bias. Test scores were normalised using participant demographic information entered into the program, including age, education, parental education, sex, ethnicity and occupation. The administration, calculation and validation of the NIH Toolbox cognitive scores have been described previously [33]. All assessments other than the Trail Making Task were delivered using an iPad (6th generation; Apple Inc., Cupertino, CA, USA). The Trail Making Task was administered and scored by an assessor who was blinded to group allocation. The cognitive domain, method of administration and direction of interpretation for each cognitive assessment are summarised in Supplementary Table S1. Participants did not complete a separate familiarisation session or alternative test versions and had no additional exposure to the cognitive assessments between baseline and the completion of the intervention. CVR to each cognitive stimulus was calculated relative to the 30 s baseline period recorded immediately before the corresponding cognitive task. Total CVR to cognitive stimuli was determined by summating the CVR across the individual cognitive tasks.

### 2.7. Data Capture and Processing for Cerebrovascular and Respiratory Outcomes

Beat-to-beat measurements of MCAv were recorded using QL Reader software, version 3.3 (Compumedics DWL, Singen, Germany) at a sampling frequency of 100 Hz and stored for subsequent offline analysis. Where a bilateral signal could not be obtained, analysis was performed using data from the available side. The MCAv data were normalised and analysed using a customised MATLAB program, version R2024b, and validated using Curve Expert Professional software, version 2.7 (Hyams Development, Chattanooga, TN, USA) to determine peak MCAv and resting MCAv. CVR was calculated using equations described previously [12]. CVR to hypercapnia and CVR to cognitive stimuli were processed and analysed by separate research assistants who were independent of participant recruitment, group allocation and primary data collection. Respiratory data were also processed and analysed independently of the primary assessor. The processed cerebrovascular and respiratory data were subsequently cross-checked by the senior researcher to ensure the accuracy and consistency of data processing.

### 2.8. Cardiovascular Function

Systolic and diastolic blood pressure, mean arterial pressure, heart rate, cardiac output, cardiac index, large and small arterial compliance, systemic vascular resistance and total vascular impedance were measured non-invasively using an HDI/PulseWave^TM^ CR-2000 Research Cardiovascular Profiling System (Hypertension Diagnostics, Eagan, MN, USA). Four consecutive measurements were recorded approximately 5 min apart using an appropriately sized blood pressure cuff positioned over the left brachial artery. The first measurement was used to familiarise participants with the procedure and was subsequently discarded. The mean of the remaining three measurements was used for analysis.

### 2.9. Anthropometric Measures

Participants were instructed to wear light clothing and remove their shoes before anthropometric measurements. Body mass was measured to the nearest 100 g using an electronic scale (Tanita Ultimate Scale 2000; Tanita Corporation, Tokyo, Japan). Height was measured to the nearest 1 cm using a wall-mounted telescopic stadiometer (Seca 220; Vogel & Halke, Hamburg, Germany). Waist and hip circumferences were measured to the nearest 1 cm using a standard measuring tape. Height, body mass, and waist and hip circumferences were measured in duplicate, and the mean of the two measurements was used for analysis. BMI was calculated as body mass in kilograms divided by height in metres squared. Waist-to-hip ratio was calculated by dividing waist circumference by hip circumference.

### 2.10. Exercise Performance and Handgrip Strength

Participants completed a 6 min walk test (6MWT) and a 60 s sit-to-stand test in accordance with published guidelines [34]. Handgrip strength was determined using hand dynamometry, as previously described [35]. Participants completed three attempts with their dominant and non-dominant hands. The first reading for each hand was used to familiarise participants with the procedure, and then discarded, and the average of the second and third readings were used for analysis. The 6MWT and 60 s sit-to-stand test were used to assess exercise performance, while handgrip dynamometry was used to assess muscular strength [35,36].

### 2.11. Statistical Analysis

Statistical analyses were performed using GraphPad Prism, version 10 (GraphPad Software, Boston, MA, USA). An initial power calculation was performed specifically for the present study using total CVR to cognitive stimuli data reported in previous nutritional and exercise intervention studies [12,31]. The calculation did not adopt the power calculations reported in those studies or the earlier intervention studies cited by them. Based on an SD of approximately 5% points derived from the nutritional intervention study and the exercise study, the power calculation indicated that 16 participants per group were required to detect a 5% point between-group difference in CVR to cognitive stimuli, with a two-sided alpha level of 0.05 and statistical power of 80%. To account for an anticipated attrition rate of 20%, we aimed to recruit 20 participants per group.

The normality of continuous data was assessed using the Shapiro–Wilk test. Baseline differences between groups for the participant characteristics presented in Table 1 were assessed using independent-samples *t*-tests or Mann–Whitney U-tests for normally and non-normally distributed continuous data, respectively. Between-group differences in longitudinal outcome measures were analysed using two-way analyses of variance to determine the effects of intervention (treatment vs. placebo), time (baseline vs. 12 weeks) and the intervention × time interaction. MCAv and respiratory variables measured at rest and peak hypercapnia were analysed using three-way analyses of variance to determine the effects of intervention, time and condition (rest vs. peak hypercapnia), and their interactions. Significant interaction effects were followed by planned pairwise comparisons between groups using the Bonferroni method. This approach was applied to the primary and secondary outcomes, including the anthropometric, mood, handgrip strength, exercise performance and cardiovascular outcomes reported in Table 2. Post hoc baseline-adjusted sensitivity analyses were performed for individual cognitive outcomes that demonstrated significant intervention × time interactions alongside significant differences in baseline group means. Week 12 values were included as the dependent variable, treatment group as the fixed factor and the corresponding baseline value as a covariate. The homogeneity-of-regression-slopes assumption was assessed by testing the baseline × group interaction. Adjusted between-group differences are reported with 95% confidence intervals. Statistical significance was set at *p* < 0.05. Data are presented as means ± SD. Effect sizes were determined using Cohen’s *d* for the primary outcomes and post hoc comparisons arising from significant intervention × time interactions using the following thresholds: ≤0.19 = trivial, ≥0.2 ≤0.49 = small, ≥0.5 ≤0.79 = medium, and ≥0.8 = large.

## 3. Results

### 3.1. Participant Flow, Baseline Characteristics and Intervention Compliance

**Figure 1:**
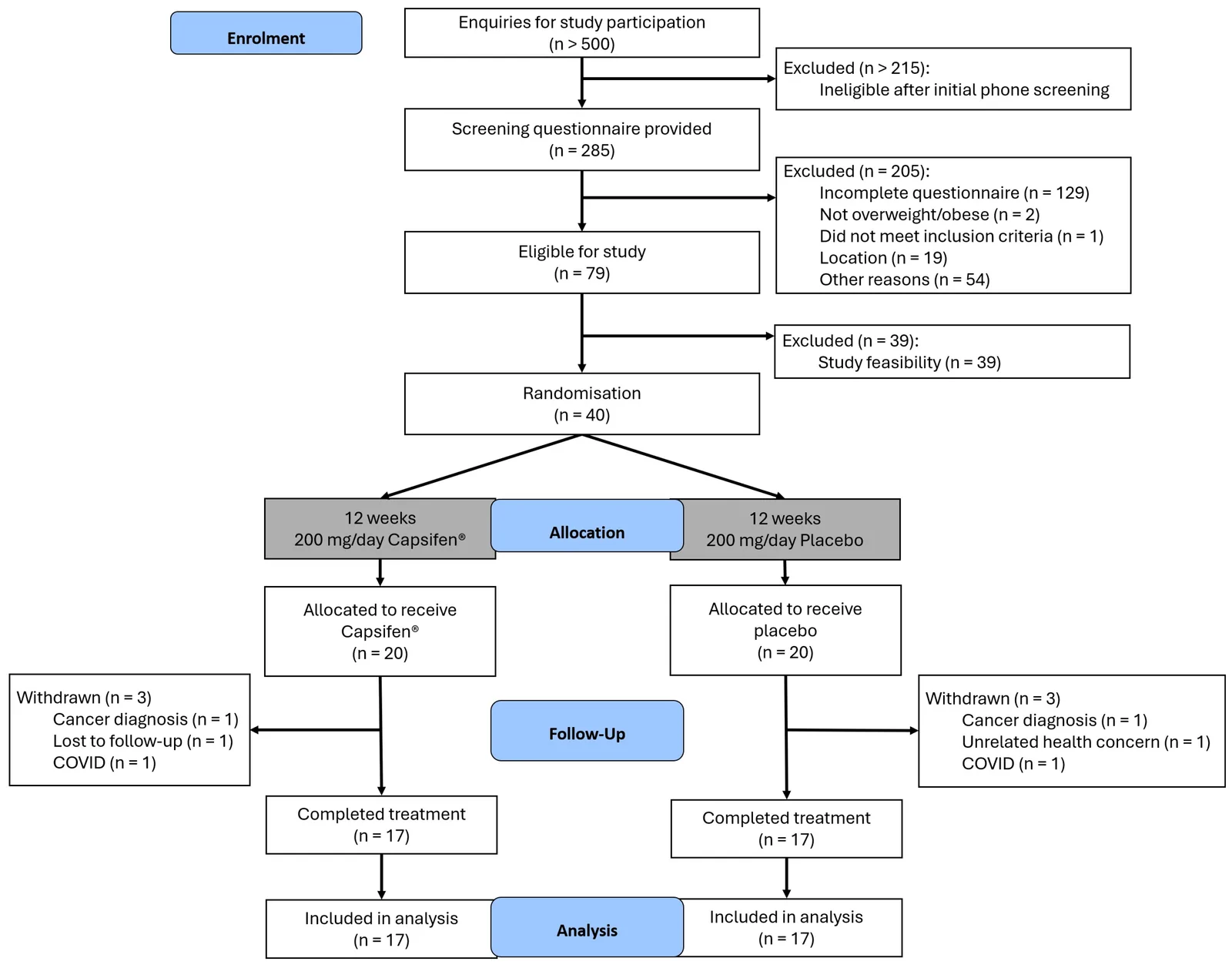
CONSORT flow diagram showing participant screening, enrolment and randomisation to 12 weeks of Capsifen^®^ or placebo supplementation, follow-up and inclusion in the final analysis.

Figure 1 shows the CONSORT participant flow diagram. More than 500 enquiries were received, and 284 individuals were provided with the screening questionnaire. Following screening, 79 individuals were eligible to participate. Of these, 39 were excluded because of study feasibility constraints, and 40 participants were randomised to either the placebo group (*n* = 20) or treatment group (*n* = 20). Six participants withdrew during the intervention for reasons unrelated to the study. Three participants withdrew from the placebo group because of a cancer diagnosis (*n* = 1), COVID-19 (*n* = 1) or loss to follow-up (*n* = 1). Three participants withdrew from the treatment group because of a cancer diagnosis (*n* = 1), COVID-19 (*n* = 1) or an unrelated health concern (*n* = 1). Thirty-four participants completed the intervention and were included in the final analysis, comprising 17 participants in each group. Participant baseline characteristics are presented in Table 1. There were no significant differences between groups at baseline for age, height, body mass, BMI or years of education (all *p* > 0.05). The distribution of males and females was identical between groups. Overall intervention compliance was 93 ± 7%, with no significant difference between the treatment and placebo groups (93 ± 6% vs. 93 ± 8%, respectively; *p* = 0.903).

### 3.2. Anthropometric Measures, Mood, Handgrip Strength, Exercise Performance, Cardiovascular Function and Biomarker Analysis

Anthropometric measures, mood, handgrip strength, exercise performance and cardiovascular function are presented in Table 2. General biochemistry, lipid profiles and hs-CRP are presented in Table 3. There was a main effect of intervention for depression scores (*p* = 0.048). However, Bonferroni-adjusted pairwise comparisons showed no significant differences between the treatment and placebo groups at Week 0 (*p* = 0.1045) or Week 12 (*p* = 0.1670). There was no main effect of time or intervention × time interaction for depression scores (*p* = 0.798). There were no intervention × time interactions for the remaining mood outcomes.

Waist circumference and waist-to-hip ratio decreased in the treatment group compared with the placebo group following the intervention (intervention × time interaction; *p* = 0.001, *d* = 0.19, and *p* = 0.021, *d* = 0.58, respectively). There were no intervention × time interactions for body mass, BMI, hip circumference or the remaining anthropometric outcomes.

Dominant and non-dominant handgrip strength increased in the treatment group compared with the placebo group following the intervention (intervention × time interaction; *p* = 0.005, *d* = 0.07, and *p* = 0.014, *d* = −0.16, respectively). Exercise performance also increased in the treatment group compared with the placebo group, as demonstrated by an increase in 6MWT distance and the number of repetitions completed during the 60 s sit-to-stand test (intervention × time interaction; both *p* < 0.0001; *d* = −0.51 and 0.86, respectively).

**Table 2:** Anthropometric, mood, handgrip strength, exercise performance and cardiovascular function at week 0 and week 12 for placebo and treatment groups.

Variable	Placebo (*n* = 17)		Treatment (*n* = 17)		*p* Value		
	Week 0	Week 12	Week 0	Week 12	Intervention	Time	Intervention × Time
Anthropometrics							
Body mass (kg)	90.3 ± 14.5	89.8 ± 14.1	94.7 ± 19.6	94.1 ± 20.1	0.468	0.251	0.957
Body mass index (kg/m^2^)	32.3 ± 4.9	32.2 ± 4.7	33.5 ± 5.7	33.3 ± 5.8	0.534	0.348	0.982
Waist circumference (cm)	109 ± 13	110 ± 13	110 ± 15	108 ± 14	0.859	0.174	**0.001**
Hip circumference (cm)	117 ± 14	116 ± 12	120 ± 12	119 ± 11	0.470	0.083	0.527
Waist-to-hip ratio	0.94 ± 0.09	0.96 ± 0.08	0.92 ± 0.10	0.90 ± 0.09	0.182	0.155	**0.021**
Profile of Mood States							
Tension	5.5 ± 2.8	6.6 ± 3.9	6.7 ± 2.6	6.1 ± 4.7	0.738	0.761	0.319
Depression	3.9 ± 3.6	4.1 ± 6.6	9.6 ± 8.6	9.1 ± 10.4	**0.048**	0.926	0.798
Anger	3.8 ± 4.4	4.3 ± 4.2	4.1 ± 6.5	6.3 ± 5.6	0.486	0.239	0.468
Fatigue	8.0 ± 4.2	10.8 ± 6.7	8.9 ± 4.7	8.9 ± 6.9	0.776	0.263	0.240
Confusion	7.3 ± 3.4	6.3 ± 4.4	8.4 ± 5.1	8.8 ± 4.7	0.176	0.808	0.513
Vigour	16.7 ± 5.5	15.5 ± 7.5	15.6 ± 7.4	13.1 ± 6.3	0.450	0.120	0.551
Total Mood	11.9 ± 16.8	16.7 ± 23.5	22.9 ± 20.8	26.1 ± 32.5	0.194	0.387	0.863
Grip strength							
Dominant hand (kg)	34.2 ± 10.3	32.8 ± 10.1	31.5 ± 10.1	32.6 ± 9.4	0.690	0.677	**0.005**
Non-dominant hand (kg)	31.7 ± 8.5	29.9 ± 8.0	31.5 ± 12.7	32.5 ± 13.0	0.755	0.506	**0.014**
Exercise performance							
Six-minute walk test distance (m)	504 ± 92	457 ± 98	451 ± 71	505 ± 91	0.946	0.692	**<0.0001**
60 s sit-to-stand (repetitions)	28 ± 5	24 ± 6	23 ± 5	28 ± 5	0.963	0.363	**<0.0001**
Cardiovascular function							
Heart rate (beats/min)	67 ± 18	73 ± 9	72 ± 12	71 ± 10	0.649	0.290	0.250
Cardiac output (L/min)	5.2 ± 0.7	5.1 ± 0.8	5.0 ± 0.8	5.1 ± 0.9	0.688	0.595	0.204
Cardiac index (L/min/m^2^)	2.6 ± 0.2	2.6 ± 0.3	2.5 ± 0.2	2.6 ± 0.3	0.845	0.177	0.847
Systolic blood pressure (mmHg)	141 ± 16	137 ± 13	142 ± 15	134 ± 16	0.846	**0.014**	0.352
Diastolic blood pressure (mmHg)	80 ± 8	78 ± 7	78 ± 10	76 ± 11	0.418	0.073	0.850
Mean arterial pressure (mmHg)	104 ± 12	101 ± 11	103 ± 12	99 ± 13	0.665	0.116	0.807
Large arterial compliance (mL/mmHg × 10)	10.8 ± 2.9	11.8 ± 4.1	11.5 ± 3.7	12.2 ± 3.3	0.610	0.160	0.836
Small arterial compliance (mL/mmHg × 10)	3.7 ± 1.4	4.4 ± 1.8	3.9 ± 1.5	5.1 ± 2.3	0.370	0.009	0.468
Systemic vascular resistance (dyne/sec/cm-s)	1642 ± 219	1610 ± 248	1672 ± 285	1545 ± 350	0.861	0.032	0.195
Total vascular impedance (dyne/sec/cm-s)	178 ± 33	163 ± 62	174 ± 56	154 ± 38	0.645	0.058	0.746

Values are presented as mean ± standard deviation. Values in bold represent significance.

**Table 3:** Biochemistry and hs-CRP analysis at week 0 and week 12 for placebo and treatment groups.

Variable	Placebo (*n* = 17)		Treatment (*n* = 17)		*p* Value		
	Week 0	Week 12	Week 0	Week 12	Intervention	Time	Intervention × Time
Sodium (mmol/L)	142 ± 2	141 ± 2	141 ± 3	141 ± 2	0.327	0.286	0.298
Potassium (mmol/L)	4.4 ± 0.3	4.5 ± 0.3	4.5 ± 0.2	4.4 ± 0.2	0.795	0.499	0.073
Chloride (mmol/L)	105 ± 2	106 ± 3	106 ± 2	106 ± 1	0.249	0.463	0.380
Bicarbonate (mmol/L)	28 ± 2	27 ± 2	27 ± 1	26 ± 2	0.274	0.153	0.351
Other anions (mmol/L)	12 ± 3	13 ± 2	12.2 ± 2.8	12.7 ± 2.1	0.879	0.064	0.308
Glucose (mmol/L)	5.6 ± 0.8	5.6 ± 0.6	5.4 ± 0.7	5.2 ± 0.4	0.242	0.112	0.235
Urea (mmol/L)	5.5 ± 1.6	5.7 ± 1.5	5.8 ± 1.1	5.7 ± 1.5	0.790	0.945	0.494
Creatinine (µmol/L)	77 ± 18	78 ± 17	75 ± 16	74 ± 15	0.584	0.550	0.443
Estimated glomerular filtration rate (mL/min)	80 ± 11	78 ± 10	81 ± 10	80 ± 9	0.622	0.158	0.844
Uric acid (mmol/L)	0.4 ± 0.0	0.4 ± 0.0	0.3 ± 0.1	0.3 ± 0.1	0.313	0.792	0.693
Total bilirubin (μmol/L)	9 ± 3	9 ± 3	9 ± 4	9 ± 3	0.926	0.519	0.860
Alkaline phosphatase (U/L)	78 ± 16	78 ± 18	76 ± 20	75 ± 18	0.635	0.537	0.687
Gamma-glutamyl transferase (U/L)	24 ± 11	22 ± 10	28 ± 13	27 ± 13	0.217	0.127	0.908
Alanine aminotransferase (U/L)	27 ± 16	24 ± 12	28 ± 17	27 ± 14	0.668	**0.014**	0.491
Aspartate aminotransferase (U/L)	24 ± 6	22 ± 5	25 ± 11	23 ± 9	0.798	**0.022**	0.850
Lactate dehydrogenase (U/L)	186 ± 28	190 ± 28	203 ± 81	183 ± 43	0.754	0.363	0.234
Corrected calcium (mmol/L)	2.3 ± 0.1	2.4 ± 0.1	2.3 ± 0.1	2.4 ± 0.1	0.847	0.242	0.749
Phosphate (mmol/L)	1.1 ± 0.1	1.1 ± 0.1	1.1 ± 0.2	1.1 ± 0.2	0.379	0.939	0.591
Total protein (g/L)	68 ± 3	69 ± 4	68 ± 5	68 ± 3	0.830	0.605	0.279
Albumin (g/L)	44 ± 3	45 ± 2	43 ± 2	43 ± 2	0.239	0.279	0.178
Globulins (g/L)	25 ± 3	24 ± 2	25 ± 3	25 ± 3	0.528	0.572	0.936
Total cholesterol (mmol/L)	4.9 ± 1.0	4.8 ± 0.8	4.8 ± 0.6	4.9 ± 0.8	0.910	0.941	0.337
Triglycerides (mmol/L)	1.9 ± 1.9	1.4 ± 0.8	1.2 ± 0.8	1.1 ± 0.6	0.151	0.261	0.536
High-density lipoprotein (mmol/L)	1.5 ± 0.4	1.5 ± 0.4	1.5 ± 0.4	1.5 ± 0.4	0.737	0.372	0.400
Low-density lipoprotein (mmol/L)	2.8 ± 0.9	2.7 ± 0.6	2.7 ± 0.7	2.8 ± 0.7	0.916	0.617	0.226
High-sensitivity C-reactive protein (mg/L)	2.8 ± 2.8	2.9 ± 2.6	3.1 ± 2.3	3.0 ± 2.0	0.805	0.846	0.539

Values are presented as mean ± standard deviation. Values in bold represent significance.

Systolic blood pressure decreased across the intervention period (main effect of time; *p* = 0.014), with no intervention × time interaction. Small arterial compliance increased and systemic vascular resistance decreased across the intervention period (main effect of time; *p* = 0.009 and *p* = 0.032, respectively), with no intervention × time interactions. There were no intervention × time interactions for the remaining cardiovascular outcomes.

There were main effects of time for alanine aminotransferase and aspartate aminotransferase (*p* = 0.014 and *p* = 0.022, respectively), with both decreasing across the intervention period. However, there were no intervention × time interactions for any general biochemistry, lipid profile or hs-CRP outcome.

### 3.3. Cerebrovascular Responsiveness to Hypercapnia

**Figure 2:**
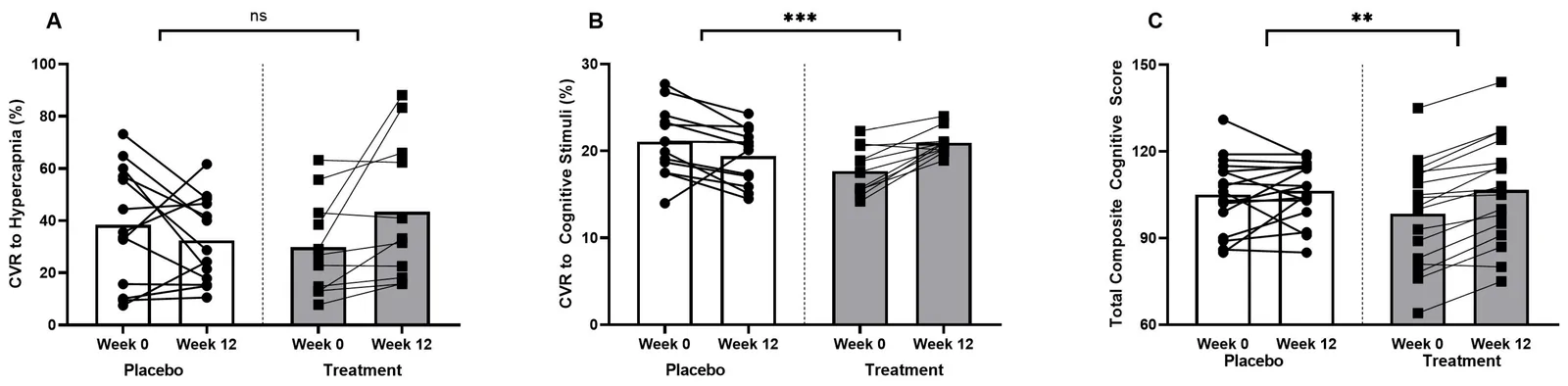
Cerebrovascular responsiveness (CVR) to hypercapnia (**A**), total CVR to cognitive stimuli (**B**) and total composite cognitive score (**C**) at week 0 and week 12 for the placebo and treatment groups. Significant difference between groups: ** *p* < 0.01; *** *p* < 0.005; ns, *p* > 0.05.

CVR to hypercapnia and the associated cerebrovascular and respiratory variables are presented in Figure 2 and Table 4. There were no main effects of intervention or time and no intervention × time interaction effects for CVR to hypercapnia.

MCAv and maximum P_ET_CO_2_ increased significantly from rest to peak hypercapnia (main effect of condition, *p <* 0.01), confirming that the 5% carbon dioxide stimulus elicited the expected physiological (respiratory and cerebrovascular) responses to the hypercapnic stimulus. There were no intervention-related differences in these responses over the intervention period. Resting breathing frequency decreased across the intervention period (main effect of time; *p* = 0.007), with no intervention × time interaction. There were no significant intervention × time interactions for the remaining cerebrovascular or respiratory variables.

### 3.4. Cognition and Cerebrovascular Responsiveness to Cognitive Stimuli

Cognition and CVR to cognitive stimuli are presented in Figure 2 and Table 5. Total cognition increased across the intervention period (main effect of time; *p* = 0.005), with no main effect of intervention (*p* = 0.5321). There was a significant intervention × time interaction for total cognition (*p* = 0.0085, *d* = 0.96). The post hoc comparison remained significant following Bonferroni adjustment (*p* = 0.0452), with total cognition higher in the treatment group than in the placebo group following the intervention.

For the individual cognitive outcomes, there were significant intervention × time interactions for List Sorting Working Memory (*p* < 0.0001, *d* = −0.95) and completion times for Trail Making Task Parts A and B (*p* < 0.0001, *d* = 0.95, and *p* < 0.001, *d* = 0.20, respectively). Bonferroni-adjusted post hoc comparisons remained significant for List Sorting Working Memory (*p* = 0.0227) and Trail Making Task Part A (*p* = 0.0409), with higher working memory scores and shorter completion times, respectively, in the treatment group following the intervention. The post hoc comparison for Trail Making Task Part B was not significant following Bonferroni adjustment. Pattern Comparison Processing Speed and Picture Sequence Memory scores increased across the intervention period (main effect of time; *p* = 0.043 and *p* = 0.005, respectively). There were also main effects of time for Trail Making Task Part A completion time and errors and Trail Making Task Part B completion time (*p* < 0.001, *p* = 0.011 and *p* = 0.013, respectively). There were no significant intervention × time interactions for the remaining cognitive outcomes.

In post hoc baseline-adjusted sensitivity analyses, List Sorting Working Memory remained significantly higher in the treatment group than in the placebo group at Week 12 (adjusted between-group difference: 3.66 points; 95% CI: 2.14 to 5.19; *p* < 0.0001). Trail Making Task Part A and Part B completion times also remained significantly lower in the treatment group (adjusted between-group differences: −12.34 s; 95% CI: −16.77 to −7.90; *p* < 0.0001, and −18.02 s; 95% CI: −28.48 to −7.55; *p* = 0.0014, respectively). The baseline × group interactions were not significant for these outcomes.

There were no main effects of intervention or time for total CVR to cognitive stimuli. However, there was a significant intervention × time interaction (*p* = 0.0002, *d* = −0.60). The post hoc comparison remained significant following Bonferroni adjustment (*p* = 0.0184), with total CVR to cognitive stimuli higher in the treatment group than in the placebo group following the intervention.

There were significant intervention × time interactions for CVR to the Flanker Inhibitory Control and Attention, Picture Sequence Memory, List Sorting Working Memory, Oral Reading Recognition and Trail Making Task Part A stimuli (*p* = 0.003, *d* = −0.37; *p* = 0.009, *d* = −0.59; *p* < 0.0001, *d* = −0.58; *p* = 0.003, *d* = −0.73; and *p* = 0.024, *d* = −0.08, respectively). However, none of the corresponding post hoc comparisons remained significant following Bonferroni adjustment. There was also a main effect of time for CVR to the List Sorting Working Memory stimulus (*p* = 0.035). There were no significant intervention × time interactions for CVR to the remaining individual cognitive stimuli.

## 4. Discussion

### 4.1. Main Findings

The aim of this study was to evaluate the effects of 12 weeks of capsaicin supplementation on cerebrovascular function and cognition in middle-aged and older adults who were overweight or obese. The main findings were that supplementation with 200 mg/day of oral Capsifen^®^ improved total cognition and CVR to cognitive stimuli compared with a placebo. In contrast, Capsifen^®^ did not improve CVR to hypercapnia. These findings indicate that capsaicin supplementation may improve cognition and cerebrovascular function during cognitive activation, reflecting improved NVC, but not CVR to hypercapnia, a physiological stimulus.

Improvements were also observed in working memory and executive function, assessed using the List Sorting Working Memory Test and Trail Making Task Part A, respectively. Although intervention × time interactions were observed for Trail Making Task Part B and CVR to several individual cognitive stimuli, these differences did not remain significant following adjustment for multiple comparisons. Capsifen^®^ supplementation also reduced waist circumference and waist-to-hip ratio and improved handgrip strength and exercise performance compared with the placebo. Together, these findings provide preliminary evidence that chronic capsaicin supplementation may improve cognition, NVC and physical function in middle-aged and older adults who are overweight or obese.

### 4.2. Cognition and Cerebrovascular Responsiveness to Hypercapnia and Cognitive Stimuli

A key finding of this study was that total CVR to cognitive stimuli increased in the treatment group compared with the placebo group following 12 weeks of supplementation. This indicates an improvement in NVC, reflecting an enhanced cerebrovascular response during periods of increased neuronal metabolism. Total cognition also increased in the treatment group compared with the placebo group, with improvements observed in working memory and executive function. These findings support preclinical evidence demonstrating that capsaicin can improve working memory and other cognitive outcomes, such as executive function [20]. The molecular mechanisms underlying the observed improvement in CVR to cognitive stimuli were not directly assessed in the present study. We speculate that one potential mechanism underlying these improvements is the activation of endothelial transient receptor potential vanilloid 1 (TRPV1) by capsaicin, which promotes the synthesis and release of NO [37,38]. Increased endothelium-derived NO results from the upregulation of eNOS and may improve endothelial function [39]. The cerebrovascular endothelium contributes to the regulation of basal tone of the cerebrovasculature and mediates acute vasodilation during periods of increased neuronal metabolism [25,39,40]. Therefore, the improvement in total CVR to cognitive stimuli observed in this study may indicate enhanced NVC and endothelial function, allowing the cerebrovasculature to respond more effectively to the metabolic demands of the cognitive tasks and consequently increase cerebral blood flow [25,39,40]. This may have contributed to the concurrent improvements in total cognition, working memory and executive function.

Although intervention × time interactions were observed for CVR to several individual cognitive stimuli, these outcomes did not remain significant following adjustment for multiple comparisons and should therefore be interpreted cautiously. However, the improvement in total CVR to cognitive stimuli remained significant following Bonferroni adjustment, supporting an overall improvement in NVC rather than an effect confined to a particular cognitive task. Similarly, intervention × time interactions were observed for List Sorting Working Memory and Trail Making Task Parts A and B. However, only List Sorting Working Memory and Trail Making Task Part A remained significant following Bonferroni adjustment. These findings provide stronger evidence for improvements in working memory and executive function, respectively, while the Trail Making Task Part B finding should be interpreted cautiously.

In contrast, there were no differences between groups in CVR to hypercapnia following the intervention. CVR to hypercapnia and CVR to cognitive stimuli assess different components of cerebrovascular function. CVR to hypercapnia reflects the capacity of the cerebrovasculature to respond to increased carbon dioxide, while CVR to cognitive stimuli reflects NVC during periods of increased neuronal metabolism. Previous research has reported improvements in CVR to hypercapnia following exercise training [12]. However, the concurrent improvement in NVC without a change in CVR to hypercapnia suggests that the cerebrovascular effects of capsaicin may be stimulus-specific. While both mechanisms act to change blood flow in the brain, they rely on different signalling pathways, distinct anatomical targets, and separate triggers. Thus, capsaicin may preferentially influence the neural, endothelial and vascular signalling involved in NVC rather than the broader cerebrovascular response to hypercapnia [17,25,39,40]. This may involve TRPV1-related central neurovascular pathways [16,17,18,19], although neural and autonomic mechanisms were not directly assessed in the present study. Further studies are required to determine the mechanisms underlying the stimulus-specific cerebrovascular response observed following chronic capsaicin supplementation.

Capsaicin may also improve cognition through its anti-inflammatory and antioxidant properties [41]. A previous study reported that 12 weeks of supplementation with 1-Isothiocyanato-6-(methanesulfinyl)hexane, a bioactive compound found in wasabi, improved working memory and episodic memory in healthy older adults [42]. Although this compound is structurally distinct from capsaicin, both are naturally occurring bioactive compounds with anti-inflammatory and antioxidant properties. The cognitive improvements observed following supplementation with 1-isothiocyanato-6-(methanesulfinyl) hexane were hypothesised to result from reductions in neuroinflammation [42]. This is consistent with preclinical evidence demonstrating that capsaicin can reduce systemic inflammation, neuroinflammation and hippocampal oxidative stress [16,17,18,19,20]. However, hs-CRP did not change following capsaicin supplementation in the present study. The persistence of excess adiposity may have maintained chronic low-grade systemic inflammation, and the 12-week intervention may have been insufficient to produce a detectable change in circulating hs-CRP, particularly in the absence of a reduction in body mass or BMI [19,37]. Baseline hs-CRP concentrations were also generally within the 1–3 mg/L range. Meta-analyses of other nutritional interventions have identified baseline hs-CRP as a potential modifier of the response to supplementation, with greater reductions observed among participants with baseline concentrations ≥3 mg/L [43,44]. Although this evidence is not specific to capsaicin, the relatively modest baseline hs-CRP concentrations in the present study may have limited the capacity to detect a measurable change. The dose or 12-week duration of Capsifen^®^ supplementation may also have been insufficient to alter systemic hs-CRP concentrations. Furthermore, hs-CRP is a general marker of systemic inflammation and may not reflect local vascular or neuroinflammatory changes [37]. Therefore, the absence of a change in hs-CRP does not exclude an anti-inflammatory mechanism, although local inflammatory and oxidative stress pathways were not directly measured in this study.

A previous study investigating acute capsaicin supplementation in young, healthy athletes reported no improvement in cognition following a single 12 mg dose [45]. However, that study is not directly comparable with the present trial because it assessed the immediate effects of a single dose in a younger, healthy population using a single cognitive assessment. Familiarisation with cognitive assessment may also have influenced the acute study findings, as familiarity can affect cognitive test responses [46]. In contrast, the present study evaluated the effects of 12 weeks of capsaicin supplementation in middle-aged and older adults who were overweight or obese using a comprehensive cognitive battery alongside concurrent assessments of cerebrovascular function. Although repeated cognitive testing can produce practice effects, the significant intervention × time interaction and Bonferroni-adjusted post hoc comparison for total cognition indicate that the observed improvement was unlikely to be explained solely by repeated testing. To our knowledge, this is the first human trial to investigate the effects of chronic capsaicin supplementation on cognition and CVR to both physiological and cognitive stimuli. Further studies are required to confirm these findings and determine the mechanisms underlying these improvements.

### 4.3. Secondary Findings

This study extended beyond the primary outcomes by examining anthropometric, cardiovascular, physical function and biomarker outcomes in middle-aged and older adults who were overweight or obese. At baseline, participants displayed characteristics consistent with metabolic syndrome, including central adiposity, elevated blood pressure and impaired metabolic health [47]. This is important because metabolic syndrome and chronic low-grade systemic inflammation are associated with cardiovascular disease and may exacerbate age-related reductions in cerebrovascular function and cognition [37,38].

Capsifen^®^ supplementation reduced waist circumference and waist-to-hip ratio compared with the placebo, despite no change in body mass or BMI. Waist circumference and waist-to-hip ratio are indicators of abdominal adiposity and cardiovascular risk and are important components of metabolic health [48]. However, these anthropometric findings should be interpreted cautiously because regional and visceral adiposity were not directly assessed in the present analysis. Furthermore, hs-CRP remained unchanged, and endothelial function was not directly assessed. Further studies should investigate whether chronic capsaicin supplementation alters specific indices of body-fat distribution, including android, gynoid and visceral adiposity, and determine whether changes in these measures are associated with improvements in cardiovascular risk, cerebrovascular function and cognition.

Capsifen^®^ supplementation also improved exercise performance, as indicated by increased 6MWT distance and 60 s sit-to-stand repetitions compared with the placebo. The 6MWT provides an assessment of functional exercise capacity, while the 60 s sit-to-stand test reflects lower-body functional capacity and muscular endurance [36]. Previous human studies have reported that acute capsaicin supplementation can increase time to exhaustion during high-intensity intermittent and resistance exercise, reduce 1500 m running time and improve upper-limb exercise performance [45,49]. Preclinical studies in animal models have also reported increased time to exhaustion during swimming following capsaicin supplementation [15,16,30]. However, human studies have found that acute capsaicin supplementation did not improve kicking, jumping or high-intensity continuous exercise performance [45,50]. These findings suggest that the effects of capsaicin may differ according to exercise modality, population, dose and duration of supplementation.

The effects of capsaicin on exercise performance may involve activation of TRPV1 and associated changes in nociception, perceived exertion or tolerance of physical discomfort [49]. However, these mechanisms were not directly assessed in the present study. The reductions in waist circumference and waist-to-hip ratio occurred concurrently with improved functional capacity. Although the absence of changes in body mass or BMI indicates that the improvements in exercise performance were not attributable to overall weight loss, reduced abdominal adiposity may support improved physical function [51]. Reduced perceived breathlessness or increased tolerance of physical exertion may also provide potential explanations for these improvements [13]. Future studies should assess perceived exertion, breathlessness, pain tolerance and cardiorespiratory fitness to elucidate the mechanisms through which chronic capsaicin supplementation may influence exercise performance. Dominant and non-dominant handgrip strength also improved in the treatment group compared with the placebo group. However, the corresponding effect sizes indicated modest favourable changes, and these findings should be interpreted cautiously. Although capsaicin may influence neuromuscular function or tolerance of physical discomfort through TRPV1-related pathways [16,17,18,19,49], the present study did not assess muscle activation, muscle quality or the mechanisms underlying the changes in handgrip strength. Further research is required to determine whether chronic capsaicin supplementation produces reproducible improvements in muscular strength and whether these changes contribute to improved physical function in middle-aged and older adults.

### 4.4. Limitations, Strengths and Future Directions

A primary limitation of this study was the relatively small sample size. This can lead to Type II errors and may have resulted in some between-group pairwise comparisons not remaining statistically significant following intervention × time interaction effects. A further methodological limitation is that TCD measures MCAv rather than volumetric cerebral blood flow and assumes that the diameter of the insonated artery remains relatively stable during measurement. Accordingly, the observed increase in CVR to cognitive stimuli should be interpreted as improved TCD-derived NVC. Nevertheless, task-related changes in MCAv are an established method for assessing NVC, consistent with previously established methodology [30] and a recent systematic review and consensus agreement [52]. Moreover, the study included middle-aged and older adults who were overweight or obese but did not have diagnosed cognitive impairment or neurodegenerative disease. Therefore, the findings may not be generalisable to individuals with a genetic predisposition to neurodegenerative disease or those with an existing diagnosis. Although improved NVC may be relevant to maintaining cognition during ageing, the present findings cannot be extrapolated to Alzheimer’s disease or other neurodegenerative conditions because these populations were not included in the study. Dietary intake was not assessed throughout the intervention because of incomplete participant reporting. Although individuals with a high habitual intake of chilli or chilli-containing supplements were excluded and the groups were matched for age, sex and BMI, changes in dietary intake during the intervention cannot be excluded as a potential influence on the findings.

Despite these limitations, this is the first human trial to examine the effects of chronic capsaicin supplementation on cerebrovascular function and cognition. The randomised, double-blind, placebo-controlled design, high intervention compliance and independent processing of cerebrovascular and respiratory data strengthen the findings. The use of a comprehensive cognitive battery alongside concurrent assessments of CVR to both physiological and cognitive stimuli also provided a more complete evaluation of cerebrovascular function and cognition. The findings demonstrate the feasibility of assessing the effects of chronic capsaicin supplementation on the human cerebrovasculature and cognition and provide preliminary evidence that findings from preclinical studies may be translatable to humans.

Further studies with larger sample sizes and longer follow-up periods are required to confirm these findings and determine the optimal dose and duration of capsaicin supplementation. Future trials should assess dietary intake throughout the intervention and include individuals with diagnosed cognitive impairment or neurodegenerative disease and those with a genetic predisposition to these conditions. Genetic markers such as the apolipoprotein E ε4 allele could be incorporated to determine whether genetic risk modifies the effects of capsaicin supplementation [53]. As participants did not have diagnosed cognitive impairment and functional outcomes were not assessed, the clinical significance of the observed improvements in cognitive scores remains uncertain. Such studies could also determine whether improvements in cognition, memory, executive function and physical capacity translate into improvements in functional independence and quality of life [6].

Oxidative stress pathways were not directly measured in this study, which limits our ability to understand the direct, upstream effects of Capsifen^®^ supplementation on antioxidant-mediated neuroprotection. Additional mechanistic outcomes, including local and systemic inflammatory markers, oxidative stress, endothelial function and autonomic regulation, may help determine the pathways through which capsaicin influences NVC and cognition. Longer-term studies are also required to establish whether these improvements are sustained and whether chronic capsaicin supplementation can slow age- and obesity-related reductions in cerebrovascular function and cognition.

## 5. Conclusions

This is the first registered trial to evaluate the effects of chronic capsaicin supplementation on cerebrovascular function and cognition in middle-aged and older adults who were overweight or obese. Twelve weeks of supplementation with 200 mg/day of oral Capsifen^®^ improved total cognition and NVC compared with the placebo but did not alter CVR to hypercapnia. Improvements were also observed in working memory and executive function, alongside reductions in waist circumference and waist-to-hip ratio and improvements in exercise performance. These findings demonstrate that chronic capsaicin supplementation is feasible in this population and provide preliminary evidence that capsaicin may improve cognition and cerebrovascular function, specifically NVC. The absence of a change in CVR to hypercapnia suggests that the cerebrovascular effects of capsaicin may be stimulus-specific. Larger and longer-duration trials are required to confirm these findings, determine the optimal dose and duration of supplementation, and establish whether the observed improvements are sustained and translate into improvements in functional independence and quality of life.

## Figures and Tables

**Table 1:** Participant baseline demographic characteristics. Values are provided as mean ± SD.

Variable	Placebo (*n* = 20)	Treatment (*n* = 20)	*p* Value
Age (years)	62.2 ± 7.6	63.2 ± 8.3	0.644
Sex (male/female)	8/12	8/12	-
Height (cm)	168 ± 7	169 ± 10	0.217
Body mass (kg)	91.3 ± 14.8	95.8 ± 18.9	0.558
Body mass index (kg/m^2^)	32.5 ± 4.8	33.5 ± 5.3	0.558
Education (years)	19 ± 4	17 ± 4	0.176

**Table 4:** Cerebrovascular and respiratory variables at rest (normocapnia) and during hypercapnia at week 0 and week 12 for the placebo and treatment groups.

Variable	Placebo (*n* = 17)		Treatment (*n* = 17)		*p* Value		
	Week 0	Week 12	Week 0	Week 12	Intervention	Time	Intervention × Time
Cerebrovascular variables							
MCAv at rest (cm/s)	41 ± 7	46 ± 14	44 ± 10	42 ± 9	0.936	0.527	0.136
Peak MCAv during hypercapnia (cm/s)	56 ± 11	59 ± 15	58 ± 19	61 ± 13	0.805	0.363	0.940
Respiratory variables							
P_ET_CO_2_ at rest (mmHg)	28 ± 4	29 ± 5	26 ± 5	27 ± 5	0.534	0.332	0.672
P_ET_CO_2_ during hypercapnia (mmHg)	36 ± 9	39 ± 8	32 ± 11	36 ± 9	0.286	0.332	0.876
Tidal volume at rest (L)	1.7 ± 0.5	1.8 ± 0.6	1.6 ± 0.9	1.8 ± 1.2	0.902	0.524	0.903
Tidal volume during hypercapnia (L)	1.3 ± 0.4	1.7 ± 0.6	1.6 ± 0.7	1.8 ± 0.7	0.279	0.168	0.569
Breathing frequency at rest (breaths/min)	15 ± 3	12 ± 4	15 ± 4	13 ± 6	0.766	**0.007**	0.476
Breathing frequency during hypercapnia (breaths/min)	13 ± 4	13 ± 5	14 ± 5	13 ± 4	0.862	0.225	0.496
Minute ventilation at rest (L/min)	18 ± 8	25 ± 12	20 ± 6	21 ± 8	0.769	0.578	0.135
Peak minute ventilation during hypercapnia (L/min)	20 ± 10	27 ± 10	22 ± 8	21 ± 6	0.490	0.219	0.094
MCA_V_/P_ET_CO_2_ (cm/s/mmHg)	1.6 ± 0.8	1.9 ± 0.3	1.5 ± 1.3	2.2 ± 1.7	0.895	0.173	0.707

Values are presented as mean ± standard deviation. Values in bold represent significance. Abbreviations: MCA_V_, middle cerebral artery velocity; P_ET_CO_2,_ partial pressure of end-tidal carbon dioxide.

**Table 5:** Cognition and cerebrovascular responsiveness to cognitive stimuli at week 0 and week 12 for the placebo and treatment groups.

Variable	Placebo (*n* = 17)		Treatment (*n* = 17)		*p* Value		
	Week 0	Week 12	Week 0	Week 12	Intervention	Time	Intervention × Time
Cognitive test							
Dimensional change card sort *	7.90 ± 0.79	8.13 ± 0.66	7.52 ± 1.94	7.75 ± 1.71	0.378	0.291	0.999
Pattern comparison processing speed *	49.77 ± 10.31	50.88 ± 10.95	49.18 ± 11.36	54.06 ± 10.81	0.710	**0.043**	0.196
Picture vocabulary test *	5.56 ± 2.21	6.17 ± 1.90	5.06 ± 2.48	5.07 ± 2.43	0.295	0.173	0.194
Flanker Inhibitory Control and Attention *	8.02 ± 0.57	7.91 ± 0.50	7.64 ± 0.93	7.70 ± 0.91	0.244	0.701	0.209
Picture sequence memory *	−1.11 ± 0.65	−0.57 ± 0.79	−1.44 ± 1.51	−0.62 ± 0.90	0.477	**0.005**	0.529
List sorting working memory *	18.53 ± 2.60	16.58 ± 2.45	15.70 ± 2.51	18.76 ± 2.13	0.666	0.152	**<0.0001**
Oral reading recognition *	5.42 ± 2.25	5.04 ± 2.17	3.96 ± 2.60	4.76 ± 2.60	0.270	0.490	0.055
Trail making task (Part A)							
Time (s)	31.65 ± 4.74	35.99 ± 5.92	42.42 ± 11.85	29.33 ± 9.03	0.424	**<0.001**	**<0.0001**
Errors made	1 ± 1	1 ± 1	1 ± 1	1 ± 1	0.908	**0.011**	0.668
Trail making task (Part B)							
Time (s)	68.13 ± 22.68	71.74 ± 18.82	85.64 ± 44.51	65.97 ± 35.60	0.585	**0.013**	**<0.001**
Errors made	2 ± 3	3 ± 7	3 ± 3	2 ± 3	0.905	0.819	0.527
Part B-Part A time difference	36.49 ± 19.72	35.76 ± 17.93	43.22 ± 36.22	36.64 ± 29.70	0.670	0.211	0.315
Cerebrovascular responsiveness (%)							
Dimensional change card sort	20.4 ± 4.7	18.1 ± 3.4	19.1 ± 4.2	20.3 ± 2.6	0.761	0.596	0.087
Pattern comparison processing speed	17.5 ± 3.7	17.2 ± 4.2	17.3 ± 3.5	19.0 ± 3.7	0.467	0.504	0.387
Picture vocabulary test	21.0 ± 4.3	20.5 ± 4.7	17.8 ± 2.7	20.2 ± 3.3	0.153	0.426	0.185
Flanker Inhibitory Control and Attention	21.0 ± 7.4	16.8 ± 5.8	16.7 ± 3.4	18.4 ± 2.3	0.500	0.196	**0.003**
Picture sequence memory	22.4 ± 6.0	20.8 ± 4.2	19.4 ± 3.8	23.0 ± 3.4	0.817	0.297	**0.009**
List sorting working memory	22.9 ± 5.3	20.8 ± 4.1	17.5 ± 3.7	22.7 ± 2.1	0.258	**0.035**	**<0.0001**
Oral reading recognition	20.5 ± 6.7	18.4 ± 4.6	15.8 ± 4.0	21.4 ± 3.4	0.131	0.656	**0.003**
Trail making task (Part A)	22.0 ± 4.7	20.7 ± 4.96	17.3 ± 4.3	21.1 ± 4.3	0.178	0.245	**0.024**
Trail making task (Part B)	20.5 ± 8.1	20.6 ± 4.2	18.4 ± 3.5	20.5 ± 3.4	0.594	0.421	0.446

Values are presented as mean ± standard deviation. Values in bold represent significance. * Normalised, computed and standardised automatically by NIH Toolbox, based on validated measures.

## Data Availability

The datasets generated during and/or analysed during the current study are available from the corresponding author upon reasonable request.

## Supplementary Materials

The following supporting information can be downloaded at https://www.mdpi.com/article/10.3390/nu18213613/s1, Table S1: Summary and interpretation of the cognitive assessments used in the study.
