# Supplementary material for: The Preliminary Effects of Capsaicin Supplementation on Cerebrovascular Function and Cognition in Middle-Aged and Older Adults Who Are Overweight or Obese
Source: Nutrients. 2026 Oct 30;18(21):3613. doi: 10.3390/nu18213613 (PMC13765294; doi:10.3390/nu18213613)
Supplement: Supplementary file 1 [file nutrients-18-03613-s001.zip › nutrients-4570283-supplementary.pdf]

Supplementary Table S1: Summary and interpretation of the cognitive assessments used in the study.

Test	Domain	Administration method	Direction of interpretation
Trail Making Task (Parts A and B)	Attention<br>Processing speed<br>Executive function	Paper	Shorter completion times and fewer errors indicate better performance
National Institute of Health Toolbox			
Dimensional Change Card Sort Test	Cognitive flexibility<br>Attention	iPad	Higher scores indicate better performance
Picture Vocabulary Test	Language<br>Crystallised cognition	iPad	Higher scores indicate better performance
List Sorting Working Memory Test	Working memory	iPad	Higher scores indicate better performance
Oral Reading Recognition Test	Language<br>Crystallised cognition	iPad	Higher scores indicate better performance
Flanker Inhibitory Control and Attention Test	Attention<br>Inhibitory control	iPad	Higher scores indicate better performance
Picture Sequence Memory Test	Episodic memory	iPad	Higher scores indicate better performance
Pattern Comparison Processing Speed Test	Processing speed	iPad	Higher scores indicate better performance
Total composite cognitive score	General cognitive function<br>General cognitive ability	iPad	Higher scores indicate better performance

Note: NIH Toolbox scores were automatically computed and normalised using participant demographic information. The administration, calculation and validation of the scores have been described previously [30,31].
